# Application and perspective of CRISPR/Cas9 genome editing technology in human diseases modeling and gene therapy

**DOI:** 10.3389/fgene.2024.1364742

**Published:** 2024-04-11

**Authors:** Man-Ling Zhang, Hong-Bin Li, Yong Jin

**Affiliations:** ^1^ Department of Rheumatology and Immunology, The Affiliated Hospital of Inner Mongolia Medical University, Hohhot, China; ^2^ Inner Mongolia Key Laboratory for Pathogenesis and Diagnosis of Rheumatic and Autoimmune Diseases, The Affiliated Hospital of Inner Mongolia Medical University, Hohhot, China

**Keywords:** CRISPR/Cas9, diseases modeling, gene therapy, neurodegenerative diseases, cardiovascular diseases, autoimmune-related diseases, cancer

## Abstract

The Clustered Regularly Interspaced Short Palindromic Repeat (CRISPR) mediated Cas9 nuclease system has been extensively used for genome editing and gene modification in eukaryotic cells. CRISPR/Cas9 technology holds great potential for various applications, including the correction of genetic defects or mutations within the human genome. The application of CRISPR/Cas9 genome editing system in human disease research is anticipated to solve a multitude of intricate molecular biology challenges encountered in life science research. Here, we review the fundamental principles underlying CRISPR/Cas9 technology and its recent application in neurodegenerative diseases, cardiovascular diseases, autoimmune related diseases, and cancer, focusing on the disease modeling and gene therapy potential of CRISPR/Cas9 in these diseases. Finally, we provide an overview of the limitations and future prospects associated with employing CRISPR/Cas9 technology for diseases study and treatment.

## 1 Introduction

The technology of genome editing facilitates precise manipulation of DNA sequences within the gene, enabling stable modification of genetic information through targeted knockouts, insertions, and replacements. There are primarily three genome editing technologies available: zinc finger nucleases (ZFN), transcription activator like effector nucleases (TALEN), and clustered regularly interspersed short palindromic repeats (CRISPR/Cas9) (Gaj et al., 2013), and the CRISPR/Cas9 genome editing technology being the most widely employed at present.

The CRISPR/Cas9 is an acquired immune system found in most bacteria and all archaea (Wiedenheft et al., 2012), which functions to protect against phages or foreign invading agents from swallowing plasmids (Barrangou et al., 2007). By utilizing a humanized Cas9 nuclease and guided RNA (gRNA) specifically designed for target gene, this system enables precise editing of any sequence or genome of eukaryotic cells (Jinek et al., 2012), thus becoming the third-generation genome editing technology after ZFNs and TALENs. Under the guidance of specific gRNA, the Cas9 nuclease cut, replace or inserts DNA sequences of organisms to precisely achieve the intended purpose of genome reediting (Cong et al., 2013).

The CRISPR/Cas9 system possesses several advantages, including high editing efficiency, ease of operation, cost effectiveness, and diversified recognition sites, etc. (Doudna and Charpentier, 2014; Knott and Doudna, 2018). It can be used in human genome editing (Mali et al., 2013) to treat genetic defects or gene mutation diseases (Wang et al., 2020; Weber et al., 2020; Zhang et al., 2020); editing plant genes to create more resilient plants (Seth and Harish, 2016; Soyk et al., 2017); eliminating pathogens by deleting their genes, such as the genomes of bacteria or viruses, in order to eliminate infectious diseases, offers hope for a complete cure for such diseases (Ebina et al., 2013; Yin et al., 2017; Ling et al., 2020).

The CRISPR/Cas9 system has been rapidly developed in all areas of the life science, is causing a seismic change in biomedical research, and is sweeping biology laboratories around the world with its remarkable efficiency and operational simplicity. Widely regarded as the most successful genome editing tool, we review the relevant research of CRISPR/Cas9 genome editing technology in some human diseases (focusing on neurodegenerative diseases, cardiovascular diseases, autoimmune diseases, and cancer) in recent years, and analyzed current applications and future perspective of CRISPR/Cas9 in these diseases.

## 2 Feature and mechanism of CRISPR/Cas9

**FIGURE 1:**
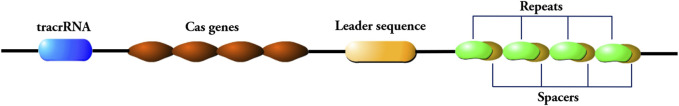
Components of the CRISPR/Cas9 genome editing system. The CRISPR/Cas9 system consists of a trans-activating crRNA (trancrRNA) region, a Cas gene region, and a CRISPR array region. The CRISPR array region consists of repeat sequences and spacer sequences. The repeat sequence is connected to the leader sequence adjacent to the 5′end of the locus, and the 5′end of the leader sequence is connected to the Cas gene region.

According to the different CRISPR/Cas sites, CRISPR systems can be divided into six different types, and the commonly used CRISPR/Cas9 genome editing system is the Type II CRISPR system, with three functional regions on the CRISPR site: The trans activating CRISPR RNA (trancrRNA) region, the different Cas genes region, and the CRISPR array region, three functional regions are linearly arranged on a single DNA strand (Figure 1). The Cas gene region is used to guide the formation of nucleases that cut DNA sequences, while the CRISPR array region contains repeat sequences and spacer sequences, and the spacer sequences will be inserted for transcription when foreign genes invade (Gupta et al., 2019).

**FIGURE 2:**
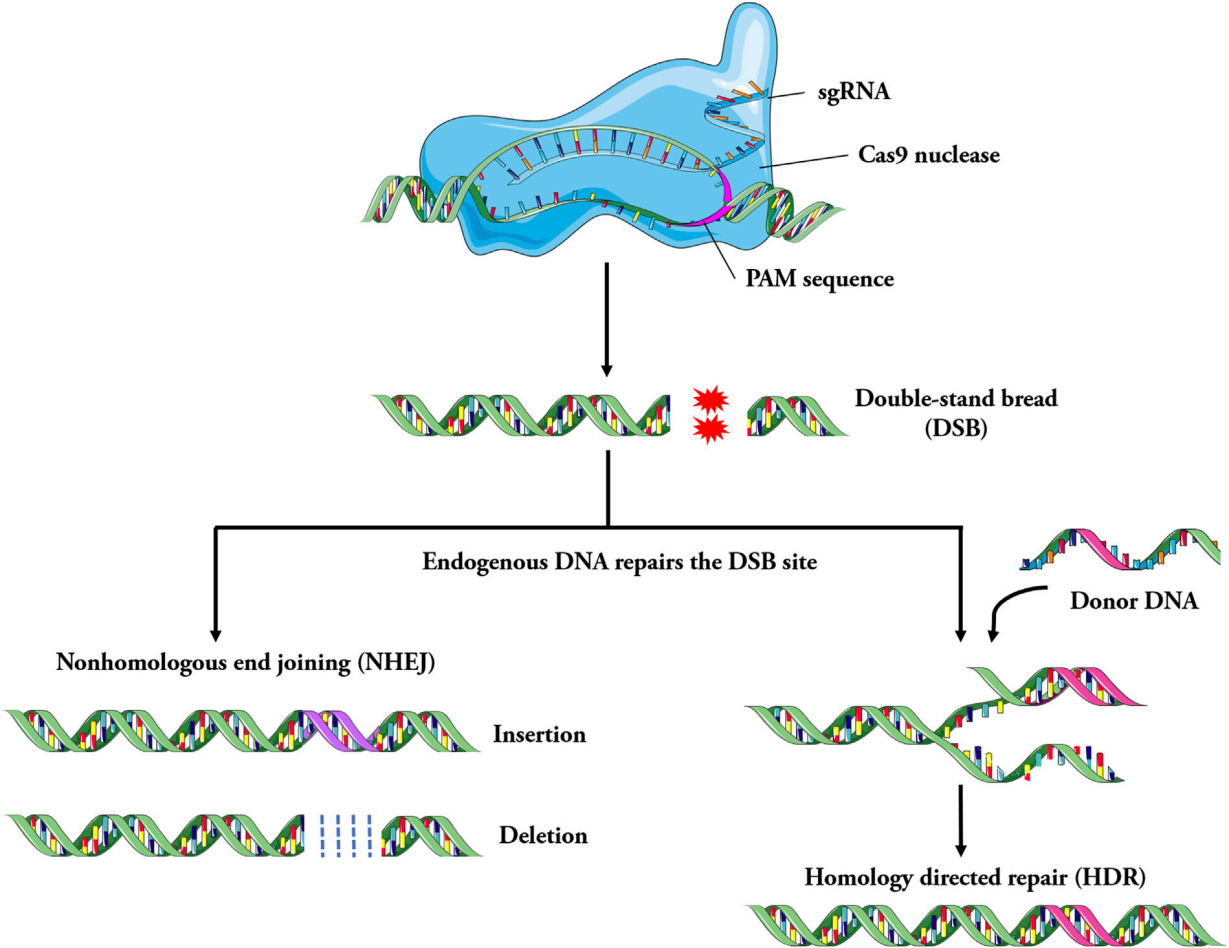
Schematic of the mechanism involved in CRISPR/Cas9 system. Specially designed sgRNA (guide RNA), that match the genomic DNA sequence containing the mutation, bind to the Cas9 endonuclease to form the Cas9-sgRNA complex. Under the guidance of sgRNA, Cas9 searches for target DNA and cut it, then double strand breaks (DSB) are generated, it leads to the activation of DNA repair mechanism. The genome is repaired by non-homologous end joining (NHEJ) or homologous recombination repair (HDR). In general, the imprecise NHEJs cause the insertion and deletion of bases, resulting in the change of the open reading frame, while the more precise HDR systems can lead to achieve site-specific repair of genes, such as disease-causing point mutations.

In the CRISPR/Cas9 system, matured CRISPR RNA (crRNA) combines with trans activating crRNA (tracrRNA) to form sgRNA (Deltcheva et al., 2011; Jiang and Doudna, 2017). The sgRNA forms a complex with the Cas9 protein. Cas9 protein is guided with sgRNA through base complementary pairing to bind to the specific locus of the genome. Subsequently, the Cas9 protein recognizes the protospacer adjacent motif (PAM) of the target gene and starts to cut the target DNA at the third base upstream of the PAM, producing double strand break (DSB) (Zaboikin et al., 2017; Iyer et al., 2019). The genome that produces DSB is subsequently repaired through either non-homologous end joining (NHEJ) or homologous recombination (HR) (Anzalone et al., 2020) (Figure 2). The NHEJ repair is imprecise, which can cause the insertion and deletion of bases, resulting in the change of the open reading frame. HR repair is precise and can achieve site specific repair of genes when homologous templates are introduced (Maruyama et al., 2015; Yeh et al., 2019).

At present, Cas9 from S. yogenes (SpCas9) is the most common CRISPR/Cas9 effector protein. Natural Cas protein still has many limitations in editing efficiency and specificity, which significantly affect its practical application. Researchers have developed different Cas9 variants to achieve better editing effect and reduce off-target activity. Previous studies have shown that two catalytic domains in Cas9, RuvC and HNH, are responsible for the cleavage of one DNA strand, respectively. By inactivating one of the domains, Cas9 nickase (nCas9) that catalyzes single strand breaks can be generated (Ran et al., 2013). Compared with wild-type Cas9 protein, the use of nCas9 can reduce off-target effects (Shen et al., 2014). Yin et al. (Yin et al., 2022a) fused SpCas9 with the optimized human recombinant protein TREX2 to generate a new gene editing enzyme, Cas9TX, which can inhibit structural abnormalities such as chromosomal translocations during gene editing and significantly improve the safety of gene editing. Qi et al. mutagenized the catalytic nuclease domain of the Cas9 protein. Two mutations were introduced to obtain a catalytically inactive Cas9 protein, also known as “dead Cas9” (Qi et al., 2013). Unlike Cas9, dCas9 does not cause irreversible changes in the genome, but only affects transcription at the target site, resulting in reversible gene silencing. It has also been shown that the fusion of dCas9 to the FokI domain reduces off-target cleavage (Guilinger et al., 2014; Tsai et al., 2014; Aouida et al., 2015; Nakagawa et al., 2015; Havlicek et al., 2017; Saifaldeen et al., 2020). The tool simultaneously binds two dCas9-FokI monomers at the target site to trigger DSBs, and the probability of two adjacent dCas9-FokI fusion molecules to bind to the off-target site nonspecifically is significantly lower than that of single molecule binding (Aouida et al., 2015; Nakagawa et al., 2015). This minimizes the risk of off-target effects. Data from Aouida et al. suggest that fdCas9 endonuclease variant is a superior platform for genome editing applications in eukaryotic systems, including mammalian cells (Aouida et al., 2015) and that all engineered fdCas9 variants have demonstrated promising gene editing activity in human cells thus far (Havlicek et al., 2017; Saifaldeen et al., 2020). The development of various Cas variants has significantly enriched the CRISPR editing tools, and improved the editing efficiency and accuracy.

The research and application of the CRISPR/Cas9 system in human diseases encompass the establishment of *in vivo* disease models, *in vitro* cell models research and gene therapy, etc. The change of open reading frame caused by NHEJ repair can be used to establish gene knockout model, while gene replacement through homologous recombination holds potential for achieving gene therapy.

## 3 CRISPR/Cas9 genome editing for neurodegenerative diseases modeling

Neurodegenerative diseases (NDs) are a broad category of diseases caused by the degeneration of neurons (Dugger and Dickson, 2017). Aging is the primary risk factor for NDs, and patients are often accompanied by cognitive decline and motor dysfunction, which can lead to severe disability or even death (Wyss-Coray, 2016). Common examples of NDs include Alzheimer’s disease, Parkinson’s disease, and Huntington’s disease. The correlations between genetic mutations and neurodegenerative diseases, and the combination of CRISPR/Cas9 with other technologies, have enabled the creation of various animal models that mimic genetic defects and provide important insights into their pathogenesis (Yang et al., 2021; Yin et al., 2022b). In fact, the construction of animal disease models also holds significant implications for investigating other diseases. Hence, we summarize the various disease animal models constructed by CRISPR/Cas9 technology (Supplementary Table S1), highlighting the respective strengths and limitations of each model in accurately recapitulating disease pathology. The focus of this section will be on the research progress and recent applications status of the CRISPR/Cas9 system in several common neurodegenerative diseases.

### 3.1 Alzheimer’s disease (AD)

AD is the most common NDs, and the neurotoxic accumulation of amyloid β-protein (Aβ) and hyperphosphorylation of Tau protein to form double helix filament are the main driving factors (Lanctot et al., 2017). Animal models play a crucial role in studying AD pathogenesis and developing treatments. However, most of the previous AD mouse models did not exhibit significant neurodegeneration (De Plano et al., 2022). CRISPR/Cas9 technology was used to create new AD models and show more accurate disease phenotypes (Lu et al., 2021). Bart De Strooper et al. established an animal model of AD using CRISPR/Cas9 technology by introducing three independent point mutations (G676R, F681Y and R684H) of APP gene. They transformed endogenous mouse APP genes into human versions and established a humanized animal model. This will help to reveal novel mechanisms of disease pathogenesis (Serneels et al., 2020). Tan et al. used CRISPR/Cas9 to introduce a deficiency in the translational start codon of exon one of the Mapt gene encoding Tau protein in a new animal model that was produced in the pure C57Bl/6J background (Tan et al., 2018).

The onset and progression of the majority of AD cases are sporadic, and in fact, only in a small number of familial AD cases (<1%), mutations in related genes lead to the production of amyloid precursor protein (APP), which promotes APP processing to produce Aβ. Nevertheless, dysregulation of Aβ metabolism is involved in both sporadic and familial AD, suggesting that limiting Aβ production may offer a treatment independent of the disease (Bhardwaj et al., 2022). Studies using the CRISPR/Cas9 system have been reported to help knock out Swedish APP mutations in patient derived fibroblasts and found a 60% reduction in Aβ, the editing efficiencies of the two designed sgRNAs for APP^SW^ in cells were 34.3% and 14%, respectively (Gyorgy et al., 2018). Sun et al. targeted the C-terminal of endogenous APP gene via CRISPR/Cas9, the robust editing of APP was observed in human iPSC derived neurons (81.8%) and mouse brains, with no detectable off-target effects. Their study shifted the processing of APP from amyloidosis related APP-β cleavage to upregulated neuroprotective APP-α cleavage, thereby reducing the production of Aβ, and showed that this method was effective in reducing the production of Aβ (Sun et al., 2019).

### 3.2 Parkinson’s disease (PD)

PD, the second most common NDs, is caused by degeneration, loss of dopaminergic neurons in the substantia nigra of the midbrain or the formation of Lewy bodies (Karimian et al., 2020). Like AD, most cases of PD are sporadic. Mutations in genes encoding alphasynuclein, PINK1, Parkin, LRRK2, and others have been found in 10%–15% of familial PD cases. The editing of classical PD related genes in mice has not yielded successful results (Qu et al., 2023), and the simultaneous knockout of three Parkinson’s disease related genes, including Parkin/PINK1/Dj-1, did not result in any observed neurodegenerative phenotype in mice (Kitada et al., 2009). The researchers constructed large animal models that were more closely resembled humans in size and physiology. Zhou et al. transfected the CRISPR/Cas9 expression vector targeting PARK2 and PINK1 into Porcine fetal fibroblasts (PFF) (Zhou et al., 2015), and successfully constructed a PD model pig by somatic cell nuclear transfer (SCNT). Zhu et al. used CRISPR/Cas9 combined with nuclear transplantation (SNCT) technology to breed a Bama mini pig PD model expressing SNCA gene E46K, H50Q and G51D mutations (Zhu et al., 2018). However, the PD model pigs did not exhibit significant neuronal loss, while the primate models appeared to be better at reproducing typical Parkinson’s pathological features (Yang et al., 2019; Li et al., 2021a; Yang et al., 2022).

CRISPR/Cas9 technology has also been used for the deletion of mutant gene expression or direct restoration of known PD causing mutations. The A53T mutation of the SNCA gene is one of the most studied mutations in PD, and rats carrying the A53T mutation and overexpression of alpha synuclein exhibit a phenotype resembling PD, which can be prevented through CRISPR/Cas9 mediated deletion of the mutant gene (Yoon et al., 2022). Furthermore, LRRK2 mutation represents the most common genetic cause of both sporadic and familial PD. Qing et al. successfully reduced the incidence of familial and sporadic PD by editing mutant LRRK2 using CRISPR/Cas9, thereby presenting a potential avenue for treating both forms of PD, they performed unbiased detection of the top 8 potential off-target sites predicted by the COD algorithm, and Sanger sequencing analysis showed no off-target indels (Qing et al., 2017).

### 3.3 Huntington’s disease (HD)

HD is a fatal ND characterized by severe psychiatric symptoms and cognitive impairment (Bates et al., 2015), which is usually inherited in an autosomal dominant manner and is primarily caused by abnormal triple amplification of CAG (cytosine adenine guanine) in the Huntington (HTT) gene on chromosome 4p16.3 (Yang et al., 2020a; Tabrizi et al., 2020). This amplification is translated into a polyglutamine (polyQ) repeat in the disease protein Huntington (HTT) (Dianov and Hubscher, 2013). The amplification of polyQ causes HTT to misfold and accumulate in the patient’s brain, resulting in preferential loss of spinous neurons in the striatum. Various transgenic mouse models of HD have been established, and it has been found that N-terminal fragments of mutant HTT with amplified polyQ repeats can accumulate in the brain, affecting motor and neuronal function (Crook and Housman, 2011; Farshim and Bates, 2018). However, in these mouse models, there was no significant selective loss of mid-spinous neurons when HD knockout (Crook and Housman, 2011). In 2018, Yan et al. used CRISPR/Cas9 technology to introduce 150 CAG repeats into endogenous porcine HTT gene and successfully generated the first porcine HD model. Notably, when the full length mutant HTT carrying 150Q was expressed endogenously in the HD porcine model, it causes significant selective neurodegeneration and movement disorders, which effectively generalizes the typical pathological and clinical features of HD patients (Yan et al., 2018).

Considering its primary genetic cause, HD is considered an ideal ND for the application of CRISPR/Cas9 technology, and studies have suggested that reducing the production of mutant HTT alleles could be an effective therapeutic approach (Ekman et al., 2019; Lopes et al., 2020). Furthermore, Jing et al. inhibited the expression of endogenous Hsp70 binding protein in the striatum of HD140QKI mice by CRISPR/Cas9, and the accumulation of HTT was significantly reduced, which indicated that CRISPR/Cas9 could play a certain role in the treatment of Huntington’s disease (Jing et al., 2021). Therefore, the removal of the mutated HTT protein has important therapeutic implications for HD, and identifying a treatment for any one neurological disease will have broad implications for the development of treatments for a large number of patients with neurological diseases.

## 4 CRISPR/Cas9 for cardiovascular diseases modeling and gene therapy

Cardiovascular disease is one of the chronic diseases that serious threaten human life caused by the combination of genes and environment (Morton et al., 2022), its incidence is rising year by year, and the proportion of population deaths is quite high, and it has become the primary disease that threatens human health (McKenna and Judge, 2021). CRISPR/Cas9 genome editing technology has ushered in a new era of cardiovascular disease research and offering the possibility for genetic correction of this ailment (Liu and Olson, 2022).

Inherited heart diseases, such as congenital heart disease (CHD), hypertrophic cardiomyopathy (HCM), and Duchenne muscular dystrophy (DMD), are caused by mutations in either a single gene or multiple genes (Austin et al., 2019; Yadav et al., 2019). There has been a lack of relatively reliable models of heart disease (Strong and Musunuru, 2017), and now with the aid of CRISPR/Cas9 technology, researchers have developed corresponding *in vivo* and *in vitro* models to study hereditary heart diseases. In the *in vivo* disease models, Alankarage et al. created CRISPR/Cas9 gene edited mouse models for functional analysis of novel missense variants identified from patients with congenital heart disease (CHD), revealing the effects of multiple congenital abnormalities on CHD (Alankarage et al., 2020). Johansen et al. used CRISPR/Cas9 and AAV9 mediated delivery of short guide RNAs targeting 3 genes (Myh6, Sav1, and Tbx20) critical for cardiac physiology to generate cardiomyocyte specific Cas9 mice, and demonstrated that Cas9 expression did not affect cardiac function or gene expression (Johansen et al., 2017). Liu et al. used CRISPR/Cas9 genome editing technology to verify the dual genes that cause hypoplastic left heart syndrome (HLHS) in mice, indicating that HLHS can be inherited through combination mode, thus providing a new paradigm for the complex genetics of congenital heart disease (Liu et al., 2017a). Other heart disease models constructed by researchers using CRISPR/Cas9 in recent years are shown in Supplementary Table S1. In the *in vitro* cell models, Li et al. used CRISPR/Cas9 technology to knock out MLP (striated muscle associated protein) in H9 human embryonic stem cell line to generate the corresponding defects type (Li et al., 2019), which later developed into HCM. Jaffre et al. successfully established the HCM model of Noonan syndrome on patient derived cardiomyocytes by using CRISPR/Cas9 generated isogenic control iPSC derived cardiomyocytes (Jaffre et al., 2019).

CRISPR/Cas9 technology is also commonly used to study the role of different genes or proteins in the mechanism of inherited heart diseases. Chang et al. constructed two FHL2 gene knockout ES cell lines using CRISPR/Cas9 technology (Chang et al., 2018), which could be used for mechanism research and drug screening of dilated cardiomyopathy (DCM). Xie et al. applied CRISPR/Cas9 gene editing technology to generate hESC related to TOF (tetralogy of Fallot), revealing the myocardial hypertrophy derived from human embryonic stem cells through HB-EGF signal transduction by rare mutations of TOF (Xie et al., 2019). Li et al. used CRISPR/Cas9 technology to create RAD (Ras associated with diabetes) deficient human ES cell lines, demonstrating that elevated intracellular calcium levels and abnormal calcium regulation were the core mechanisms of RAD deficiency leading to cardiac hypertrophy (Li et al., 2020). Roche et al. used CRISPR/Cas9 to introduce Brugada syndrome (BrS) mutations in human induced hiPSC-CMs, which, as a genetic background independent of patients, revealed the mechanism of sodium channel inactivation triggering BrS (de la Roche et al., 2019). By constructing PLN mutant hiPSC-CM cell line, Smith et al. confirmed that the mutation could lead to delayed response of cardiomyocytes to β-receptor agonists (Smith et al., 2018). Ang et al. combined iPSC with CRISPR/Cas9 technologies to establish a congenital heart disease model related to GATA4 gene mutation *in vitro*, aiming at exploring the pathogenesis caused by this gene mutation (Ang et al., 2016).

CRISPR/Cas9 shows promise as a potential treatment for cardiovascular diseases meanwhile. In the case of DMD, somatic genome editing therapy for the disease has demonstrated promising outcomes in both animal models and *in vitro* human models. Nelson et al. respectively loaded CRISPR-SaCas9 and two sgRNAs into AAV vectors and used the AAV-CRISPR gene editing system to target the deletion of exon 23 in DMD gene of MDX model mice (Nelson et al., 2019), resulting in the expression of dystrophin with normal functionality. The newborn mice injected intravenously with the AAV-CRISPR system still had higher gene editing efficiency and maintained high expression of restored dystrophin protein 1 year later. They found that the expression of SaCas9 in various types of muscle cell, while also detected genome editing events in other tissues at levels slightly above the detection limit (∼0.1%). Furthermore, deep sequencing analysis of the top 10 predicted off-target sites showed no significant increase in off-target cutting after 1 year. Amoasii et al. coupled Cas9 with sgRNA to target regions near the DMD gene exon 51 splicing receptor sites, and used adeno-associated viruses to deliver the CRISPR gene editing component to four dogs, where dystrophin recovered to 3%–90% of normal levels in the heart muscle. The dogs that received the highest dose had levels of dystrophin that were 92% of normal. The muscle tissue of treated dogs also improved (Amoasii et al., 2018), the analysis of predicted off-target sites revealed no specific off-target gene editing above background levels in treatment animals. Moretti et al. used a Cas9 mediated exon excision method to restore the DMD reading frame, which enabled short but functional dystrophin expression and improved bone and myocardial failure in DMD pigs, and this method prevented susceptibility of DMD cardiomyocytes to arrhythmias (Moretti et al., 2020). Although no mutations were detected at the five most likely predicted off-target sites, and no genome editing events were observed in the liver and kidneys, it is important to further evaluate possible off-target mutagenesis in longer term animal studies.

For cardiovascular diseases, CRISPR/Cas9 technology has just begun to be widely used, there is no doubt that for some cardiovascular diseases with high mortality, the application of CRISPR/Cas9 technology will be faster and more convenient to create cell and animal models, which is conducive to the research of cardiovascular development and disease pathogenesis.

## 5 CRISPR/Cas9 application for autoimmune-related diseases

The primary characteristic of autoimmune diseases is that the disorders function of the immune system, the body is unable to distinguish between its own antigens and foreign antigens, and mistakes the normal components of the body for foreign substances to attack, resulting in long term inflammation (Marrack et al., 2001). The genetic background underlying autoimmune diseases is intricate, involving multiple genes and pathways in their pathogenesis. Various genetic factors can lead to the development of autoimmune diseases such as rheumatoid arthritis, systemic lupus erythematosus, type 1 diabetes, and multiple sclerosis (Cooper et al., 1999; Lee et al., 2022). CRISPR/Cas9 provides numerous novel ideas for research and treatment of autoimmune diseases including the screening of autoimmune disease regulatory genes, the construction of disease models, and the attempt to be used in the treatment of autoimmune diseases.

The CRISPR/Cas9 system has been used to identify regulatory sequences for certain important autoimmune risk loci, such as CD69 and IL2RA, the latter of which is associated with Crohn’s disease (Huang et al., 2017b; Simeonov et al., 2017). Studies have demonstrated that the destabilization of Treg cells can promote the development of autoimmunity, primarily characterized by the downregulation of transcription factor Foxp3 and the acquisition of proinflammatory properties. Cortez et al. have developed a combined CRISPR based screening platform for primary mouse Treg cell phenotype study, using this technology to perform targeted loss of function screening analysis of approximately 500 intranuclear factors, thereby identifying gene regulatory programs that can promote or interfere with Foxp3 expression (Cortez et al., 2020). Additionally, researchers used CRISPR whole genome screening technology to perform a comprehensive search across the whole genome in mouse model of type 1 diabetes, aiming to identify modifying factors that influence the survival of islet β-cells (Cai et al., 2020).

CRISPR/Cas9 has also been used in the construction of some autoimmune disease models. Researchers used CRISPR/Cas9 technology to generate Trex1 D18N/D18N point mutant mice, which display a multitude of human autoimmune related symptoms, including significantly declined survival time and damage to various organs such as the heart and kidneys. Additionally, augmented levels of autoantibodies were observed (Xiao et al., 2019). The other mouse models of autoimmune diseases are also presented in Supplementary Table S1. There are also many studies on the application of CRISPR/Cas9 to construct *in vitro* cell models of autoimmune diseases. Sevim et al. constructed a model for familial hemophagocytic lymphohistiocytosis 2 (FHL2) in order to assess the effectiveness of coculturing with mesenchymal stromal cells as an alternative therapy when bone marrow transplantation is unavailable (Sevim et al., 2018). The autoimmune regulatory gene (AIRE) has also been investigated using CRISPR technology, revealing that disruption of adhesion between the thymic medullary epithelial cells and thymus cells is one mechanism through which AIRE interference leads to autoimmunity (Speck-Hernandez et al., 2018). Furthermore, another study employing CRISPR/Cas9 technology on A20 demonstrated that genetic variation in the A20 deubiquitinase (DUB) domain increased the risk of systemic lupus erythematosus (SLE) and rheumatoid arthritis (Odqvist et al., 2019).

Gene therapy is a promising treatment for autoimmune diseases, while there were limited researches on the use of CRISPR/Cas9 in treating such conditions (Lee et al., 2022). Nevertheless, we cannot disregard the potential application of CRISPR/Cas9 in autoimmune diseases. Differentiated immune cells can be genetically modified using CRISPR technology. For instance, a recessive mutation in IL2RA led to reduced levels of IL2 receptor α chains in FOXP3 Tregs from a patient with a family history of autoimmune diseases. After targeted modification with CRSIPR, it found that the patient’s T cells began to express normal levels of IL2RA (Roth et al., 2018), the researchers also conducted targeted loci amplification (TLA) sequencing and observed no evidence of off-target integration above the detection limit (∼1% of alleles). In relation to rheumatoid arthritis (RA), Yang et al.'s study suggests that MYC and FOXO1 genes are implicated (Yang et al., 2020b), making these genes suitable candidates for targeted treatment using CRISPR/Cas9. Additionally, CRISPR/Cas9 has also been used to modify genes that may affect FOXP3 expression, enabling predominance of Treg expression and promoting the effect of adoptive Treg therapy in rheumatoid arthritis (Safari et al., 2018).

To date, numerous studies using CRISPR/Cas9 to treat autoimmune diseases have been conducted in cells (Table 1), and reports on clinical trials involving human subjects are very limited. However, it is undeniable that CRISPR/Cas9 plays a beneficial role in the regulation of autoimmune diseases, so it is necessary to conduct further comprehensive research, particularly more extensive *in vivo* investigations.

**TABLE 1:** The cell models using CRISPR/Cas9 in autoimmune-related diseases.

Autoimmune disease	Cell lines	Target gene	Results	References
Rheumatoid Arthritis	Primary human CD4^+^ T cells	MYC, FOXO1	The MYC and FOXO1 genes may serve as the etiological factors of RA	Yang et al. (2020b)
Multiple Sclerosis	Primary human CD4^+^ T cells; lymphoblastoid cell lines	DDX39B	The epistatic interaction between DDX39B and IL7R regulates the splicing of IL7R exon 6, increasing the risk of Multiple Sclerosis	Galarza-Munoz et al. (2017)
Inflammatory Bowel Disease	subepithelial myofibroblasts (SEMF)	PTPN2	PTPN2 expression was increased in the affected ileum; deletion of PTPN2 resulted in higher levels of STAT3 and p-Erk1/2 and proliferation	Li and Kuemmerle (2019)
	colonic epithelial cells	SGK2	SGK2 protein was localized in the cytoplasm of ulcerative colitis colonic epithelial cells	Mokhtar et al. (2019)
Systemic Lupus Erythematosus	monocytes and B cells	CXorf21	CXorf21 knockdown resulted a decreased expression of TNF-α and IL-6	Harris et al. (2019)
	human U937 monocytes	TNFAIP3	The A20 C103A cells or cells carrying the rs2230926 polymorphism exhibit an increased frequency of neutrophil extracellular trap formation and produce autoantibodies targeting citrullinated epitopes	Odqvist et al. (2019)
Type 1 Diabetes Mellitus	peripheral blood mononuclear cells (PBMCs)	LCK	G allele of SNP of LCK increases the risk of type 1 diabetes mellitus	Zhu et al. (2019)

## 6 Application of CRISPR-Cas9 in cancer research

In recent years, CRISPR/Cas9 has become more widely used in cancer research and treatment, and there have been some encouraging advances, first reflected in the establishment of cancer animal models to study the pathogenesis of cancer. The creation of animal models for cancer is an important approach to studying the functional aspects associated with cancer genes. In the previous animal model establishment process, manipulation techniques were applied at the level of zygote stage to accomplish gene editing operations of embryonic stem cells with homologous recombination technology. The clinical application of CRISPR/Cas9 has outstanding simplification advantages for cancer animal models. Xue et al. using CRISPR/Cas9 technology successfully constructed a mouse model for liver cancer by simultaneously targeting tumor suppressor genes PTEN and P53 in mouse hepatocytes (Xue et al., 2014). Mou et al. injected Kirsten rat sarcoma viral oncogene (KRAS) gene templates along with sgRNA targeting tumor suppressor gene TP53 into wild-type B6 mice (Mou et al., 2019), resulted in tumors being observed in their livers 1 month later, thus successfully establishing a tumor model. Tyler et al. achieved successful construction of a mouse model for small cell lung cancer (SCLC) by simultaneously knocking out tumor suppressor genes Trp53 and Rb1 using CRISPR/Cas9 technology (Ng et al., 2020), their study provides an applicable mouse model of humanized SCLC that is suitable for studying the pathogenesis of SCLC. The construction of mouse models of other cancer using CRISPR/Cas9 technology is illustrated in Supplementary Table S1.

The findings from cellular and animal model studies suggest that CRISPR/Cas9 technology holds promise as a potentially effective approach for cancer treatment. The activation of oncogenes and the mutation of tumor suppressor genes make cancer cells expand and metastasize indefinitely, therefore, using CRISPR/Cas9 gene editing in cancer cells is expected to alter the biological characteristics of cancer cells, and provide a novel strategy for tumor treatment. Currently, several strategies using CRISPR/Cas9 for gene editing in cancer cells: 1) Introduction of tumor suppressor genes using CRISPR/Cas9 to inhibit tumor growth. Moses et al. used CRISPR/Cas9 along with trans-activator VP64p65-Rta (VPR) to activate the expression of PTEN in cancer cells, demonstrated that the activation of PTEN can significantly inhibit the downstream carcinogenic pathway (Moses et al., 2019). 2) Correction of deleterious mutations using CRISPR/Cas9 to suppress tumor development. Koo et al. used adenovirus (AdV) to deliver Cas9 protein and sgRNA targeting the EGFR oncogene with a single nucleotide missense mutation (CTG > CGG) (Koo et al., 2017). This method enabled to distinguish cancer causing mutations from wild-type EGFR alleles and precisely eliminate cancer causing mutant EGFR alleles. The frequency of EGFR mutant alleles in h1975 tumors was determined to be 50% (±5.3%), 40% (±2.8%), and 35% (±4.1%) at 7, 9, and 11 days post initial injection using Ad/sg EGFR and Ad/Cas9 vectors. Furthermore, no off-target effects were observed among the identified 17 potential off-target sites. Cheung et al. used the CRISPR/Cas9 system to target the point mutation L858R of EGFR (Cheung et al., 2018), the study used PAM to distinguish cancer mutations from normal mutations, providing high specificity in the degree of single nucleotide substitutions, and observed reduced expression of EGFR and inhibited cell proliferation in cells harboring the L858R mutation, providing novel insights into using CRISPR/Cas9 to selectively eliminate oncogenic mutations for cancer treatment. 3) Using CRISPR/Cas9 to target and knockout cancer related genes for tumors treatment, Zhen et al. used CRISPR/Cas9 to knockout E6 and E7 protein related genes that play a crucial role in the development and maintenance of cervical cancer, and the results showed that the knockout seriously damaged the survival ability of cancer cells (Zhen et al., 2016). Fusion genes are specific to cancer cells and are involved in many tumors, the researchers used CRISPR/Cas9 technology to insert the “suicide gene” HSV1-tk into the location of the MAN2A1-FER fusion gene break point, and found that the “suicide gene” can inhibit tumor growth, thus the method may be used as a viable strategy to treat tumors carrying the fusion gene (Chen et al., 2017).

Additionally, alterations in the gene expression of immune cells may reduce their ability to defend against and eliminate tumors. The utilization of CRISPR/Cas9 for gene editing in immune cells can enhance their cytotoxicity against tumors. The current strategies mainly involve: 1) Engineered T cells to eliminate tumors by knockout immune checkpoints. As an immune checkpoint, the high expression of PD-1 on T cells will inhibit the ability of T cells to initiate immune response, thereby rendering tumor cells susceptible to evading from the immune system. In 2020, Lu et al. injected CRISPR edited T cells targeting PD-1 into patients with advanced non-small-cell lung cancer (NSCLC). Clinical research results indicated a gene editing efficiency ranging from 8.7% to 31.2%, accompanied by significant reduction in PD-1 expression levels, thereby demonstrating that clinical application of CRISPR/Cas9 edited T cells is generally safe and feasible (Lu et al., 2020). Dong et al. found that DHX37 also serves as a crucial immune checkpoint (Dong et al., 2019), and knocking out DHX37 in CD8-positive T cells can enhance the effectiveness of adoptive immunotherapy against triple-negative breast cancer. 2) Using CRISPR/Cas9 to modify the antigen recognition capability of CAR-T cells. CAR-T cell immunotherapy employs genetic engineering to modify T lymphocytes to express chimeric antigen receptors that capable of identifying tumor cells and activating T cells simultaneously. Traditional CAR-T therapy exhibits certain defects, such as the limited number of T cells to be treated and the short *in vivo* survival duration. Stadtmauer et al. modified CAR-T cells with CRISPR/Cas9, demonstrated that edited CAR-T cells could persist for 9 months in patients with blood related cancers and maintain stable cytotoxicity compared to less than 7 days survival observed in original CAR-T cells (Stadtmauer et al., 2020).

The current research on utilizing CRISPR/Cas9 for gene editing of cancer cells to target tumors is primarily in the stages of basic research, and its direct application in clinical treatment remains limited. Clinical trials involving CRISPR/Cas9 are predominantly conducted *in vitro*, necessitating the isolation of cells from patients. Subsequently, the CRISPR/Cas9 system is employed for gene correction before being reintroduced into the patient. This process is influenced by various factors such as delivery systems and editing efficiency. However, with continuous advancements in gene editing technology, it is anticipated that CRISPR/Cas9 will possess significant potential to advance both cancer research and treatment.

## 7 Limitations of CRISPR/Cas9 in disease treatment and application

Although CRISPR/Cas9 is a well-established gene editing tool and has been extensively utilized in the research and treatment of various diseases, its clinical application is still in its early stages. There are several challenges that need to be addressed, including off-target effects, delivery methods, efficiency, and safety.

Off-target effects are a limitation of CRISPR/Cas9 technology and are considered a significant risk in in vivo gene therapy (Du et al., 2023). These effects occur when Cas9 proteins bind to PAM-like sequences or when guide RNAs (gRNAs) bind to unexpected nucleotide sequences at the target site. It has been reported that their efficiency exceeds 50% (Zhang et al., 2015). Off-target cleavage can result in unintended editing of normal genes, potentially disrupting their function, causing undesirable mutations, cytotoxicity, and even diseases. Selecting and designing the optimal sgRNA sequence is crucial to minimize or eliminate off-target effects. Studies have demonstrated that the structure of sgRNA influences its targeting ability, and increasing the GC content at the end of the sgRNA sequence’s PAM (Protospacer adjacent Motif) improves its targeting efficiency (Ren et al., 2014). Additionally, shortening the length of the sgRNA sequence enhances its sensitivity to residual target DNA mismatches, significantly reducing off-target effects (Hazafa et al., 2020). Furthermore, the development of high fidelity SpCas9 variants is considered the most promising strategy to address off-target effects in the context of enhanced Cas9 protein (Kulcsar et al., 2020). It is important to note that off-target effects remain unresolved *in vivo* and are closely associated with delivery techniques. Most clinical trials have focused on gene editing *in vitro* using patient derived cells, this approach reduces the risk of off-target effects and metastatic challenges but may not be suitable for all diseases.

To enhance the specificity of gene editing and achieve effective and precise therapy, it is crucial for CRISPR/Cas9 components to overcome various physical barriers and directly enter target cells (Xu et al., 2021). Additionally, the gene editing process necessitates the simultaneous delivery of functional Cas9 protein and sgRNA into the nucleus (Chen et al., 2020). Therefore, the delivery method plays a crucial role in CRISPR/Cas9 mediated editing therapy. Previously, physical and viral vectors have been explored for delivering CRISPR/Cas9 components. Physical delivery methods such as microinjection, electroporation, and hydrodynamic drug delivery exhibit high application efficiency *in vitro* but do not meet the requirements for *in vivo* applications (Liu et al., 2017b). Viral vectors also face limitations in their transformation into clinical grade therapeutic agents, including immunogenic reactions, limited packaging capacity, off-target effects, and high production costs (Tong et al., 2019; Xu et al., 2019). Non-viral vectors based on nanotechnology and materials science have been utilized in cancer therapy due to their low immunogenicity, high biocompatibility, and optimal payload capacity (Parra-Nieto et al., 2021). However, during the delivery process, CRISPR/Cas9 loaded nanoparticles encounter blood barriers, including degradation of CRISPR/Cas9 components by various enzymes in plasma (Kim et al., 2011), the clearance of nanocarriers by mononuclear phagocyte system or macrophages (de Lazaro and Mooney, 2021), and the filtration of nanocarriers by glomeruli (Han et al., 2014). Compared to the gene editing efficiency *in vitro*, the *in vivo* CRISPR/Cas9 editing efficiency is significantly lower, partially due to the low delivery efficiency of nanocarriers (Xu et al., 2021). Therefore, the lack of a safe and efficient delivery system remains the major obstacle to the clinical application of CRISPR/Cas9.

The immune system’s reaction triggered by the Cas9 protein or vector in the host represents a significant challenge in the application of CRISPR/Cas9 technology (Du et al., 2023). The Cas9 protein, originating from *Streptococcus* pyogenes, is considered an external antigen that can provoke an immune response upon introduction into the body (Charlesworth et al., 2019). While extreme immune responses to Cas9 are not extensively documented, the presence of Cas9 antibodies in humans indicates the potential exacerbation of hazards associated with CRISPR/Cas9 based gene therapy (Li et al., 2021b). Researchers are increasingly focusing on the immunogenicity caused by vectors, particularly viral vectors, as humans may have been previously exposed to them (Du et al., 2023). Thus, the immune response generated by the CRISPR/Cas9 gene editing framework constitutes a primary detrimental factor in the context of *in vivo* gene therapy using CRISPR technology.

CRISPR/Cas9 is an effective gene editing tool, but it is not yet an ideal clinical treatment. CRISPR requires a higher level of safety. Although CRISPR/Cas9 mediated gene editing has shown promising results in clinical studies, more in depth studies are needed to ensure that CRISPR/Cas9 can be safely and effectively applied to treat human diseases.

## 8 Conclusion and future perspectives

The discovery and research of the CRISPR/Cas9 system has changed the process of genome editing in the field of life sciences. In comparison to the ZFN and TALEN systems, it offers some incomparable advantages. In theory, every eight bases in the genome can find a site that can be edited with CRISPR/Cas9, so the technique can operate on almost any gene. Moreover, CRISPR/Cas9 system has more scalability, CRISPR/Cas9 gene editing technology can achieve a single base editing at the level of cells and organisms (Rees and Liu, 2018; Porto et al., 2020), is the most promising gene editing technology. Furthermore, one of its key strengths lies in its easy of use nature, enabling that almost any laboratory can do the work with this system.

**FIGURE 3:**
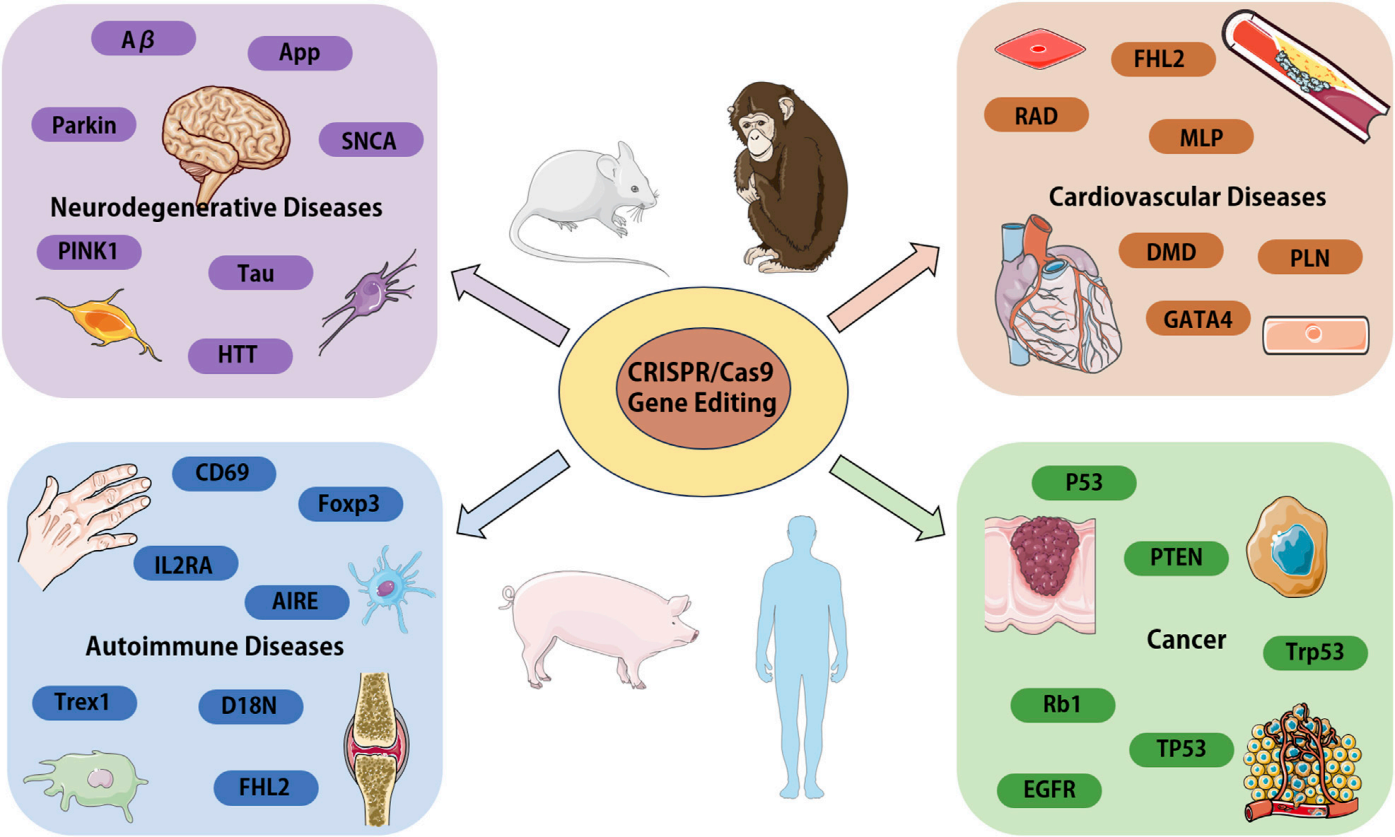
Overview of the CRISPR/Cas9 systems application in various human diseases. We focus on the genes and proteins associated with disease, which could be target for disease modeling or the candidates for gene therapy. Aβ, amyloid β-protein; APP, amyloid precursor protein; PARK2, parkin RBR E3 ubiquitin protein ligase; PINK1, PTEN-induced putative kinase 1; SNCA, a-synuclein; Tau, microtubule associated protein tau; HTT, huntington protein; FHL2, four and a half LIM domains 2; RAD, radish; MLP, membrane lipoprotein precursor; GATA4, GATA binding protein 4; DMD, Duchenne muscular dystrophy gene; PLN, phospholamban; IL2RA, interleukin 2 receptor subunit alpha; CD69, CD69 molecule; Foxp3, forkhead box P3; AIRE, autoimmune regulator; Trex1, three prime repair exonuclease 1; EGFR, epidermal growth factor receptor; p53, tumor suppressor gene; PTEN, phosphatase and tensin homolog; Trp53, transformation related protein 53; Rb1, RB transcriptional corepressor 1; TP53, tumor protein p53.

From the various research reports and literatures on CRISPR/Cas9 system in recent years, it is not difficult to find that the application and advancement of this technology will likely help people to have a deeper understanding of the pathogenesis of diseases and provide more directions for targeted treatment of diseases. In this review, we only focus CRISPR/Cas9 research applications in neurodegenerative diseases, cardiovascular diseases, autoimmune related diseases, and cancer (summarized in Figure 3). In fact, CRISPR/Cas9 researches in variety of other human diseases have been reported. Today, advocates precision medicine and personalized medicine are being promoted, the development of the technology can also better promote the implementation of such medical treatment, so as to solve a series of problems in clinical treatment. Notably, compared with traditional genome editing technology, CRISPR/Cas9 genome editing technology also highlights great advantages in the establishment of animal models, the application of this technology can ensure the accurate and time saving establishment of disease animal models, thereby greatly facilitating the development of new drugs and the study of drug mechanism of action.

Although CRISPR/Cas9 technology is widely used for gene knockout or knock-in labeling, large fragment deletion, and transcriptional regulation, and has become an important tool for exploring the pathological mechanisms of diseases. However, certain challenges still need to be addressed, including the highly concerning off-target effects, delivery system optimization (van Haasteren et al., 2020), and enhancement of gene editing accuracy and efficiency. Consequently, the clinical application of CRISPR/Cas9 technology in disease still faces certain challenges (Behr et al., 2021). However, CRISPR/Cas9 technology is constantly being improved and optimized, such as by modifying the Cas9 protein, so that it does not cut the double strand DNA, but only cut the single strand, under the guidance of a specific double sgRNA, the off-target efficiency will be greatly reduced. Therefore, in the future, CRISPR/Cas9 technology will be widely used in the mechanism research and clinical treatment of diseases, and finally overcome the treatment problems of some diseases, and bring hope to more patients.

In summary, CRISPR/Cas9 is an innovative genome editing tool with significant potential for development. With the continuous advancement of basic research and clinical applications, CRISPR/Cas9 will bring new hope for disease treatment across various fields. Genome editing technology is still developing, and its integration with disease treatment is progressively close, thus expected to make substantial contributions to human health.
