# Supplementary material for: Application and perspective of CRISPR/Cas9 genome editing technology in human diseases modeling and gene therapy
Source: Front Genet. 2024 Apr 11;15:1364742. doi: 10.3389/fgene.2024.1364742 (PMC11043577; doi:10.3389/fgene.2024.1364742)
Supplement: Supplementary file 1 [file Table1.docx]

**Table1.** The application of CRISPR/Cas9 in generating animal models of diseases.

| Disease | Type | Genetic anomaly | Species | Modification approach | Pathology and Phenotype | Pros and Cons | Reference |
| --- | --- | --- | --- | --- | --- | --- | --- |
| Neurodegenerative diseases | Alzheimer’s Disease (AD) | APP | Pig | Transgenic | No pathological changes over 5 years | This is the first study investigating memory in transgenic minipigs with the APPsw mutation for AD. No evidence for memory impairment in these transgenic pigs at relatively young ages. | Sondergaard et al. (2012) |
|  |  | PSEN1 | Pig | Transgenic | No AD‑like pathological changes over 3 years | They presented RMCE piglets with one copy of the Alzheimer’s disease-causing mutation, PSEN1M146I. This is the first time to generate transgenic mammal using minicircles and SCNT. | Jakobsen et al. (2013) |
|  |  | APP | Cynomolgus monkeys | Transgenic | Model established and ongoing observation | The generation of two transgenic cynomolgus monkeys overexpressing the human gene for APP with Swedish, Artic, and Iberian mutations. However, they used transgenic models but not knock-in models. Thus, the inserted transgene destroys endogenous gene(s) and may affect the phenotype(s). | Seita et al. (2020) |
|  |  | APP | Rat and mouse | Transgenic | No AD‑like pathological changes at 2 years of age | They created mice and rats harboring a humanized Aβ sequence in the endogenous App gene. The models produce about three times more (human) Aβ compared to the WT rodent original strains, but this is still two times less than compared to humans. An advantage of these new models is that the human Aβ ELISAs routinely used in AD research can be used to study the different Aβ species. | Serneels et al. (2020) |
|  |  | Mapt | Mouse | knockout | Mice had no overt phenotype | Mice had no overt phenotype, but, in line with previous models, they showed a significantly reduced susceptibility to excitotoxic seizures, with normal memory formation in young mice. | Tan et al. (2018) |
|  | Parkinson’s disease (PD) | PINK1/ PARK2 | Pig | knockout | No obvious neuronal loss, normal behavior | Typical symptoms of Parkinson’s disease, such as shaking, rigidity, slowness of movement, and difficulty in walking, have not been observed in 7-month-old live mutant pigs in this study. Gene-targeted pigs can be effectively achieved by using the CRISPR/Cas9 system combined with SCNT without detectable off-target effects. | Zhou et al. (2015) |
|  |  | PINK1/ Parkin /DJ-1 | Pig | knockout | No obvious neuronal loss, normal behavior | They demonstrated that the simplicity, efficiency, and power of the CRISPR/Cas9 system to allow for the modification of multiple genes in pigs and yield results of high medical value. The gene modified piglets remain healthy and display normal behavior at the age of 10 months. | Wang et al. (2016) |
|  |  | E46K/H50Q/G51D | Pig | knockout | No PD-specific pathological changes at 3 months of age | They did not observe α-synuclein-immunopositive dopaminergic neurons in the gene-edited minipigs at 3 months of the age pathology or loss of SN. | Zhu et al. (2018) |
|  |  | PINK1 | Rhesus monkeys | knockout | Severe neuronal loss, Motor function deficits | The CRISPR/Cas9-mediated large deletion with other mutations can completely eliminate PINK1 expression and function, resulting in more severe phenotypes and neuropathology, as seen in some dead newborn monkey. | Yang et al. (2019) |
|  |  | PINK1/DJ-1 | Monkey | knockout | Classic PD symptoms, severe nigral dopaminergic neuron loss | They observed age-dependent severity in Parkinsonian symptoms, loss of nigral dopaminergic neurons, and a-synuclein pathology, which demonstrated that both genetic and aging factors play important roles in the development of PD. | Li et al. (2021) |
|  |  | PINK1 | Monkey | knockout | Severe neuronal loss Motor function deficits | They demonstrated for the first time that PINK1 kinase activity rather than its mitochondrial function is selectively essential for neuronal survival in the primate brain and suggest that PINK1 kinase dysfunction may be involved in PD and other pathological conditions in humans. | Yang et al. (2022) |
|  |  | Parkin, DJ-1, PINK1 | Mouse | knockout | Normal morphology and numbers of dopaminergic and noradrenergic neurons in the substantia nigra and locus coeruleus | Inactivation of all three recessive PD genes is insufficient to cause significant nigral degeneration within the lifespan of mice, suggesting that these genes may be protective rather than essential for the survival of dopaminergic neurons during the aging process. | Kitada et al. (2009) |
|  | Huntington’s disease (HD) | HTT | Ovine | Embryonic expression of transgenic HTT | No obvious neurodegeneration, mild behavioral phenotypes | At 7 months of age, the offspring of transgenic showed a loss in DARPP32 immunoreactivity from the GP and putamen. The reduction suggests an early impairment in the regulation of the physiological state of striatal neurons via dopamine, a hallmark feature of the disease. | Jacobsen et al. (2010) |
|  |  | HTT | Mouse | knockout | impact motor and neuronal function | The mice are more likely to develop robust phenotypes within the lifetime of a mouse, as they are modeling a childhood onset, rather than a late onset, disease. | Farshim and Bates (2018) |
|  |  | HTT | Pig | Embryonic expression of transgenic N-terminal mutant HTT | No neurodegeneration and gross motor deficits | They used lentiviral infection of porcine embryos and report the successful germ line transmission through successive generations of a HD transgene encoding the first 548 aa of HTT with 124 glutamines under the control of human HTT promoter. At about one year of age, sperm number and oocyte penetration were severely affected in TgHD minipigs. | Baxa et al. (2013) |
|  |  | HTT | Pig | SCNT: Knock‑in HD‑150Q | Age-dependent neurological symptoms and Selective neurodegeneration in the striatum | They demonstrated for the first time that large mammals can recapitulate overt and selective neurodegeneration and the severe symptoms caused by the mutant protein expressed at the endogenous level. Although the pig model can more faithfully recapitulate the neurodegeneration seen in HD patients, using large animal models is not without limitations, including high animal costs, high facility expenses, and more stringent regulations. | Yan et al. (2018) |
| Cardiovascular diseases | Coronary heart disease (CHD) | G6pd | Rat | knockout | G6PDS188F mutants developed less large arterial stiffness and hypertension | They illuminated new insight into the mechanisms through which the loss of-function mutation in G6PD protects the rats from HTN—a major risk factor for coronary artery disease. | Kitagawa et al. (22020) |
|  |  | Myocd | Mouse | knockin | New mouse models for analyzing MYOCD protein expression, localization, and binding activity in vivo. | They introduced new Myocardin mouse models wherein epitope tags (3xFLAG and 3xHA) were used to independently target the last coding exon of the Myocd locus. They anticipated similarly tagged mice will be developed for rigorous protein analyses and accelerated discoveries in vascular biology. | Lyu et al. (2018) |
|  |  | PBX1 | Mouse | knockin | Congenital heart defects were observed in the homozygous mutant embryos at multiple stages of development. | They presented the first functional model of a missense PBX1 variant and provided strong evidence that p.R184P is disease-causal. They did not observe changes in expression of the PBX1 cardiac target gene, Pax3, in the R184P model. | Alankarage et al. (2020) |
|  | Hypertrophic cardiomyopathy (HCM) | Sav1, Tbx20, Myh6 | Mouse | knockout | In Vivo Genome Editing of Myh6 results in a Severe Dilated Cardiomyopathy | They generated cardiomyocyte-specific Cas9 mice and demonstrated that Cas9 expression alone does not affect cardiac function or the expression of cardiac stress markers. They targeted 3 genes critical for cardiac physiology, Myh6, Tbx20, and Sav1, only observed a cardiac phenotype in Myh6-edited mice. | Johansen et al. (2017) |
|  |  | Dnmt1 | Rat | knockout | Dnmt1 deficiency activates pathways involved in myocardial protection and anti-apoptosis in response to pathological stress. | They generated myocardium-specific Dnmt1 knockout (Dnmt1 KO) rats using CRISPR-Cas9 technology. This study first investigated in vivo the impact of genome-wide cardiac DNA methyltransferase deficiency on physiological development and the pathological processes of heart tissues in response to stress. | Wu et al. (2020) |
|  |  | Gaa | Mouse | knockin | Three-month-old mice demonstrated skeletal muscle weakness and hypertrophic cardiomyopathy but no premature mortality. | They applied CRISPR‑Cas9 homology‑directed repair (HDR) using a novel dual sgRNA approach flanking the target site to generate a Gaaem1935C>A knock‑in mouse model. A significant divergence of the model from human GAA c.1C>A IOPD is the lack of infantile mortality in KI mice. | Kan et al. (2022) |
|  |  | MYH7 | Mouse | knockin | Left ventricular posterior wall (LVPW) hypertrophy with systole in MYH7 G823E/- mice at 2 months of age. | They produced a C57BL/6N mouse model with a point mutation (G823E) at the mouse MYH7 locus with CRISPR/Cas9. The G823E variant plays an important role in the pathogenesis of HCM. | Xia et al. (2022) |
|  | Duchenne muscular dystrophy (DMD) | Dmd | Mouse | knockout | Severe muscular dystrophy phenotyp, ~50% decrease in the specific and absolute tetanic force in the EDL muscle. | They presented a simple and efficient strategy for correction of exon 44 deletion mutations by CRISPR-Cas9 gene editing in a new mouse model. They also demonstrated the importance of the dosages of these gene editing components for optimal gene correction in vivo. | Min et al. (2019) |
|  |  | Dmd | Mouse | knockout | Gradual age-dependent decrease in muscle strength, increased creatine kinase, muscle fibrosis and central nucleation. | This new model of DMD will be useful for validating therapies based on skipping exons that encode the N-terminal ABD and for improving our understanding of the role of the N-terminal domain and central rod domain in the biological function of dystrophin. | Egorova et al. (2019) |
|  |  | nSMase2/Smpd3 | Mouse | knockout | Reduced muscular degeneration and decreased inflammation responses, but later showed exacerbated muscular necrosis. | Ablation of the nSMase2/Smpd3 gene in mdx mice ameliorates membrane instability in the sarcolemma, improves muscle force and performance, and reduces excess inflammation in the early stages. Signaling pathways modulated by the nSMase2/Smpd3 protein through lipid rafts might be novel therapeutic targets for DMD. | Matsuzaka et al. (2020) |
|  | Hypoplastic left heart syndrome (HLHS) | Sap130, Pcdha9 | Mouse | knockout | F1 intercrosses yielded HLHS in double-homozygous F2 offspring. | Mutations in Sap130 and Pcdha9, genes not previously associated with CHD, were validated by CRISPR–Cas9 genome editing in mice as being digenic causes of HLHS. | Liu et al. (2017a) |
| Autoimmune-related diseases | Systemic lupus erythematosus (SLE) | TREX1 | Mouse | Knockout | Systemic inflammation that consistently recapitulates many characteristics of human AGS and SLE. | Ablation of cGas gene in the Trex1D18N/D18N mice rescued the lethality and all detectable pathological phenotypes. These results indicated that cGAS is a key mediator in the autoimmune disease associated with defective TREX1 function. | Xiao et al. (2019) |
|  |  | cGAS | Mouse | Knockout | Slightly exacerbated, and not attenuated, disease. | This study demonstrated that the cGAS-STING pathway does not promote systemic autoimmunity in murine models of SLE. These data have important implications for cGAS-STING-directed therapies being developed for the treatment of systemic autoimmunity. | Motwani et al. (2021) |
|  |  | AID | Mouse | Knockout | Significant improvements in lupus nephritis, a rebound in marginal zone B lymphocyte populations and a restoration of splenic and germinal center architecture. | They utilized CRISPR-Cas to ablate AID expression directly in the BXSB background. One limitation of this work is that only a single founder was used to develop the BXSB. | Zhu et al. (2020) |
|  | Inflammatory bowel diseases (IBD) | Sec1 | Mouse | Knockout | Exacerbated symptoms of colitis, Sec1 negatively regulated the secretion of key inflammatory factors in the intestinal environment. | Using Sec1 in mice as a unique proxy, they verified that FUT2, when expressed abnormally, contributed to the pathogenicity of IBD, by modulating human intestinal mucosal inflammation, cell proliferation and apoptosis. Sec1 may also be used as a biomarker and a new therapeutic target for IBD. | Cai et al. (2023) |
|  |  | Nod2, Atg16l1, Il23r | Rat | knockout | Continued phenotypic characterization of the models is ongoing but preliminary data shows that they faithfully recapitulate human disease. | They hypothesized that rats might be a better model species than mice to model IBD. These are the first rat models specifically generated for the study of IBD susceptibility. | Men et al. (2021) |
|  | Multiple sclerosis (MS) | CCL20, CCR6 | Mouse | Knockout | Clinical phenotypes of experimental autoimmune encephalomyelitis (EAE) in the chronic phase were slightly exacerbated | They generated CCL20-KO and CCR6-KO mice using the CRISPR/Cas9 system. CCL20/CCR6-mediated cell migration is not necessarily required for the onset of EAE, and may be compensated for by other chemokine signals. | Sachi et al. (2023) |
|  |  | HDC | Mouse | Knockout | No effect of Hdc deletion on EAE | This study refutes the role of histamine in EAE, unveils a role for neutrophil-derived histamine in IgE-mediated anaphylaxis, and establishes a new mouse model to re-explore the inflammatory and neurologic roles of histamine. | Morin et al. (2021) |
|  | Psoriasis | Zdhhc2 | Mouse | Knockout | Dramatically inhibited pathology of the ear skin following imiquimod treatment. | The functional identification of Zdhhc2 and mechanistic discoveries in this study provide an insight into possibilities of manipulating the enzymatic activity of zDHHC2 for treatment of skin inflammatory diseases such as psoriasis. | Zhou et al. (2020) |
|  |  | Mvd | Mouse | Knockin | When induced by IMQ, MvdF250S/+ mice exhibited decreased skin inflammation. | They first generated MvdF250S/+ mice and explored the difference in skin inflammation induced by external stimulation between WT and MvdF250S/+ mice, providing a new viewpoint on the biological function of MVD. | La et al. (2023) |
|  | Type 1 diabetes mellitus (DM) | MHC | Mouse | Knockout | More robust T1D development was observed | The NOD-cMHCI/II-/- mice described in the study are the ideal platform for introducing any combination of HLA class I– and II–encoding transgenes. | Racine et al. (2018) |
|  |  | Nfkbid | Mouse | Knockout | A more rapid onset of T1D in the mutant stock | Nfkbid expression levels appear to be an important contributory factor to how efficiently autoreactive diabetogenic CD8+ T-cells undergo thymic negative selection. | Presa et al. (2018) |
|  |  | Conserved non-coding sequence (CNS) of FOXP3 | Mouse | Knockout | CNS1 deletion did not alter the development of T1D or glucose tolerance. | The role of CNS1-dependent pTregs might be influenced by environmental factors. These data could provide further insight into the interplay between the immune system and environment that determines both mouse and human disease susceptibility and outcomes. | Holohan et al. (2019) |
|  |  | Human insulin | Mouse | Knockin | The incidence of diabetes is much lower in HuPI mice. | This work highlights the feasibility of using CRISPR/Cas9 to create mouse models of human diseases that express proteins pivotal to the human disease. Furthermore, it reveals that even subtle changes in proinsulin protect NOD mice from diabetes. | Elso et al. (2019) |
|  |  | Ptpn22 | Mouse | Knockin | Increased insulin autoantibodies and earlier onset and higher penetrance of T1D. | This is the first report demonstrated enhanced T1D in a mouse modeling human PTPN22R620W and the utility of CRISPR-Cas9 for direct genetic alternation of NOD mice. | Lin et al. (2016) |
| Cancer | Liver cancer | Pten, p53 | Mouse | Knockout | Simultaneous targeting of Pten and p53 induced liver tumors. | Further studies are required to fully evaluate the side effects of the CRISPR system in mice and other organisms. This study underscores the power of the CRISPR/Cas9 system for rapid genome editing and the development of novel cancer models in the mouse. | Xue et al. (2014) |
|  |  | Myc, AKT1, p53 | Mouse | Knockin and Knockout | Similar transcriptome and oncogenic signaling pathways with human cHCC-ICC. | They provided a simple GEMM for cHCC-ICC with direct clinical translational value. However, it remains elusive whether other gene combinations can lead to the cHCC-ICC formation in the same experimental setting. Moreover, the model does not exhibit the metastatic feature of cHCC-ICC, as evidenced by no lung metastatic lesion observed. | Xu et al. (2022) |
|  |  | Axin1 | Mouse | Knockout | Ablation of Axin1 alone does not lead to HCC formation, but cooperates with overexpression of c-Met to promote HCC development in mice. | As AXIN1 mutations are among the most frequent genetic events in human HCC, it would be crucial to develop murine HCC models to study targeted therapies for HCC with AXIN1 mutations. | Qiao et al. (2019) |
|  |  | PTPN22 | Mouse | knockout | Mice homozygous for this alteration (PEP-619WW) resist tumor growth. | Future studies will need to examine which PEP-619WW-bearing immune cells are necessary and/or sufficient to control tumors, as well as understand whether these results could translate into improved therapies for patients. | Orozco et al. (2021) |
|  | Lung cancer | Rb1, Trp53, Rbl2 | Mouse | knockout | Generate SCLC through somatic gene editing of Rb1, Trp53, and Rbl2 in mice. | This model can be used to interrogate candidate oncoproteins, tumor suppressors, or therapeutic targets without the need for complex and time-consuming breeding strategies. This is the first SCLC GEMM developed on a pure congenic background, which allows cell lines established from this model to be used for syngeneic transplant studies. | Oser et al. (2019) |
|  |  | p53, LKB1, KRAS | Mouse | knockout | Leading to macroscopic tumors of adeno-carcinoma pathology. | This mouse can be used in conjunction with a variety of guide RNA delivery reagents, providing an attractive model for studying the interplay of mutations in biological processes and disease. | Platt et al. (2014) |
|  |  | Eml4, Alk | Mouse | knockout | Eml4-Alk inversion drives tumor development in lungs. | CRISPR/Cas9 system can be exploited to directly engineer Eml4-Alk rearrangements in vivo in adult mice. This approach represents a simple and cost-effective method to model chromosomal rearrangements that could be used as an alternative to classical transgenic or knockin mice for a variety of biological and cancer studies. | Blasco et al. (2014) |
|  | Myeloid malignancy | Tet2, Runx1, Dnmt3a, Nf1, Ezh2, Smc3 | Mouse | knockout | Splenomegaly, leukocytosis, excess myeloid cells in the BM, circulating blasts in peripheral blood and diagnostic features of AML in the bone marrow. | By using lentiviral delivered sgRNA: Cas9 components, they engineered novel models of myeloid malignancy bearing mutations in 4–5 different genes that can be propagated and studied through serial transplantation. | Heckl et al. (2014) |
|  |  | Stk11 | Mouse | knockout | Rapid lethality with enhanced fibrosis, osteosclerosis and an accumulation immature cells in the bone marrow, as well as enhanced engraftment of primary human MPN cells in vivo. | Liver Inducible Kinase (LKB1)/Serine threonine kinase 11 (STK11) as a driver of leukemic transformation in MPN. STK11 is a more potent tumor suppressor in the MPNs. They provided insights into potential therapeutic approaches to better treat blast phase disease. | Marinaccio et al. (2021) |
|  | Sarcoma | Trp53 | Mouse | knockout | Deletion of Trp53 was required for anchorage-independent growth and to form tumors resembling sarcoma. | Sarcomas generated with CRISPR-Cas9 technology are similar to sarcomas generated with conventional modelling techniques and suggest that CRISPR-Cas9 can be used to more rapidly generate genotypically and phenotypically similar cancers. | Huang et al. (2017a) |
|  | Colorectal cancer | Apc | Mouse | knockout | Reliably produced tumors within six weeks. | CRISPR-Cas9-based in situ Apc editing in the colon epithelium induces adenoma formation. These tumors demonstrated histological features of adenomatous change and nuclear β-catenin localization, which are characteristic of human neoplasms of the colon and aberrant activation of the Wnt signaling pathway. | Roper et al. (2017) |
